# Phenotypic spectrum and genetic heterogeneity of cystic fibrosis in Sri Lanka

**DOI:** 10.1186/s12881-019-0815-x

**Published:** 2019-05-24

**Authors:** Neluwa Liyanage Ruwan Indika, Dinesha Maduri Vidanapathirana, Hewa Warawitage Dilanthi, Grace Angeline Malarnangai Kularatnam, Nambage Dona Priyani Dhammika Chandrasiri, Eresha Jasinge

**Affiliations:** 1 Department of Biochemistry, Faculty of Medical Sciences, University of Sri Jayewardenepura, Nugegoda, Sri Lanka; 2 Department of Pathology, Faculty of Medical Sciences, University of Sri Jayewardenepura, Nugegoda, Sri Lanka; 3 Department of Biochemistry and Molecular Biology, Faculty of Medicine, University of Colombo, Colombo 8, Sri Lanka; 4 Department of Chemical Pathology, Provincial General Hospital, Ratnapura, Sri Lanka; 5 Department of Chemical Pathology, Lady Ridgeway Hospital for Children, Colombo 8, Sri Lanka

**Keywords:** Cystic fibrosis, Sri Lanka, Mutations, Phenotypic features, Gene sequencing

## Abstract

**Background:**

Cystic fibrosis has been largely under-diagnosed and thus, limited data is available on the incidence of cystic fibrosis in Sri Lanka. Our aim is to describe the phenotypic and genotypic spectrum of children with cystic fibrosis in Sri Lanka.

**Case presentation:**

This report describes 10 unrelated cystic fibrosis cases with phenotypic features of cystic fibrosis and abnormal or intermediate sweat tests. The most common phenotypic features in this sample of symptomatic patients were persistent or recurrent lower respiratory tract infections, failure to thrive and Pseudo-Bartter syndrome. Altogether 7 cystic fibrosis causing mutations were identified in 10 patients. Except delta F508 which is the commonest mutation worldwide all the other mutations detected in Sri Lankan patients are rare mutations. 1161delC and V456A detected in our patients are South Asian mutations. The other mutations such as [C.1282C > G; C.2738A > G], C.53 + 1G > C, 2184insA and a deletion encompassing exons 4 to 11 have been reported previously from European patients with cystic fibrosis.

**Conclusion:**

These cases highlight the importance of considering the diagnosis of cystic fibrosis in children and young adults presenting with persistent respiratory tract infections associated with severe malnutrition and Pseudo-Bartter syndrome, especially in low income countries where newborn screening for cystic fibrosis is not available. The spectrum of *CFTR* mutations in Sri Lanka is heterogeneous and possibly linked to genetic flow from Indian subcontinent and Europe. The common mutations should be identified by sequencing the entire *CFTR* gene in adequate number of cystic fibrosis patients in order to design a mutation panel for common regional mutations.

## Background

Cystic fibrosis (CF) is an autosomal recessive disorder caused by mutations in a large gene located in chromosome number 7 encoding a protein called cystic fibrosis transmembrane conductance regulator (CFTR). The disease is common among Caucasians with an incidence of 1 in 2,500 but uncommon in Asians (1 in 31,000 live births). It is a multisystem disorder affecting epithelia of the respiratory tract, exocrine pancreas, intestine, hepatobiliary system, male genitourinary tract and exocrine sweat glands giving rise to characteristic clinical features including chronic sinopulmonary disease, pancreatic insufficiency, recurrent pancreatitis, neonatal bowel obstruction, rectal prolapse, focal biliary cirrhosis, failure to thrive (FTT), Pseudo-Bartter syndrome and male urogenital abnormalities resulting in obstructive azoospermia [1].

Although CF is diagnosed through newborn screening (NBS) programs in developed countries, there are still a large number of patients diagnosed outside of the neonatal period in low income countries where NBS for CF is not available. Early diagnosis of CF and institution of medical care can greatly improve the long-term outcome of these patients. When the diagnosis of CF is being considered outside of the NBS context, family history of CF or CF carrier status and typical signs and symptoms play an important role in defining likelihood of CF. For symptomatic non-screened populations, the same diagnostic criteria recommended for the screened population should be used to confirm CF [2, 3].

Even with recent advances in molecular diagnostics the quantitative pilocarpine iontophoresis sweat chloride test remains the gold standard confirmatory test in diagnosing CF which can accurately diagnose more than 98% of cases of CF. Sweat conductivity can be used as a screening test [4, 5]. Individuals who meet sweat chloride criteria for CF diagnosis still benefit from *CFTR* genetic testing because of the availability of CFTR-modulating therapies that are specific to certain mutations. Patients with intermediate sweat test results should undergo *CFTR* genotyping for confirmation of diagnosis. Therefore *CFTR* genotyping has become an equally important part of CF diagnosis [6].

To date, there is no NBS program for CF in Sri Lanka. Reliable incidence figures for CF, carrier rates or mutation frequencies for Sri Lankan population are not available. The first case reported from Sri Lanka in 1994 is of a child who was diagnosed based on typical clinical features and a positive agar-silver chromate test. This semi quantitative screening test was performed in the absence of facilities for standard sweat test and genetic studies [7]. Existence of CF in Sri Lanka was spotted back again through the approach of opportunistic screening of symptomatic patients using sweat test at Lady Ridgeway Hospital for Children. Thus, CF has been largely underdiagnosed in Sri Lanka due to low index of suspicion and poor availability of facilities for diagnosis.

Testing for *CFTR* mutations in a population is done by a panel test. These panels should be customized with common variants seen in the local population to have a high mutation detection rate. It is not clear as to what available mutation detection kits can be used for Sri Lankan patients other than delta F508 mutation which is so far the commonest mutation in any population. Therefore, the present study aimed at identifying common *CFTR* mutations in Sri Lankan patients, so that a molecular diagnostic test can be developed for the population. The study also describes the phenotypic spectrum of symptomatic patients with CF in Sri Lanka.

## Case presentation

The Department of Chemical Pathology of Lady Ridgeway Hospital for Children is the only laboratory which performs sweat test in Sri Lanka to date. A retrospective study was done on all patients referred to the department who were symptomatic with phenotypic features suggestive of CF. Among the observed phenotypic features were chronic or recurrent lower respiratory tract infections (LRTI), bronchiectasis, steatorrhoea, FTT and Pseudo-Bartter syndrome. Altogether 10 patients with positive sweat conductivity tests (>50 mmol/L), abnormal or intermediate sweat chloride tests and positive genetic studies were identified by examining clinical records of patients who were investigated at the department from 2012 to 2018. When more than one child is affected from a family, only the elder child was included in the case series.

Sweat was collected by Wescor Macroduct sweat collection system following stimulation by pilocarpine iontophoresis. The collection was done by a trained medical officer and assisted by a trained medical laboratory technologist adhering to the latest guidelines of Clinical Laboratory Standards Institute (CLSI) [8]. The samples were analyzed for sodium chloride levels by sweat conductivity analyzer (sweat conductivity method) and for chloride levels by Chlorocheck model 3400 Chloridometer (coulometric titration method). Sweat was analyzed soon after the collection so that the sweat was not subject to evaporation. The laboratory maintains its quality of the sweat test method by participating in Randox International Quality Assessment Scheme (RIQAS). Sweat test was interpreted according to the recently updated reference intervals given by consensus guidelines from the Cystic Fibrosis Foundation (CFF) [9]. Since the chloridometer was available only after April, 2005, the first 4 cases could not undergo sweat chloride test.

The 2017 CFF Consensus Guidelines for interpretation of sweat chloride concentration are as follows;

- ≤29 mmol/L: normal, CF unlikely (exceptions occur)
- 30-59 mmol/L: intermediate, possible CF
- ≥60 mmol/L: abnormal, indicative for diagnosis of CF

Genomic DNA from blood was sent to Foundation for Research in Genetics and Endocrinology (FRIGE), Ahmedabad, Gujarat, India to perform amplification refractory mutation system - polymerase chain reaction (ARMS-PCR) assay and restriction fragment length polymorphism technique (RFLP) in order to identify common *CFTR* mutations in India [10]. This was based on speculation that spectrum of *CFTR* mutations in Sri Lankan population would be similar to that of Indian subcontinent due to genetic flow with historical movements of populations evident by scientific literature [11–14]. This panel included common 8 *CFTR* mutations in India including delta F508, 1525-1G-A, 1161delC, 1792insA, R117H, S549 N, R553X and G551D [15, 16].

*CFTR* gene sequencing was performed initially at Canterbury Health Laboratories, Christchurch, New Zealand. The *CFTR* gene coding regions (with flanking intronic sequences) and the *CFTR* promoter region (including 410 base pairs upstream of the catabolite activator protein binding site) were amplified by PCR and analyzed by bi-directional automated DNA sequencing.

Gene sequencing was subsequently done at Centogene AG, Rostock Germany. The *CFTR* gene was analyzed by PCR and next-generation sequencing of both DNA strands of the entire coding region and the highly conserved exon-intron splice junctions. PCR-based amplicon library capture was utilized. This test was developed and its performance validated by CENTOGENE AG. MLPA (multiplex ligation-dependent probe amplification) analysis was performed using SALSA MLPA probemix P091-D1 provided by MRC-Holland to test for deletions or duplications within or including the *CFTR* gene. The identity of the genes detected by the reference probes is available online (www.mlpa.com).

We identified 10 unrelated patients with cystic fibrosis out of which 7 patients fulfill the standard diagnostic criteria given by CFF.

### Demographic and clinical characteristics

All of the patients in this group had characteristic phenotypic features suggestive of CF which are summarized in Table 1. The commonest clinical phenotype was recurrent or persistent LRTIs (100%). FTT and Pseudo-Bartter syndrome were observed in 8 (80%) and 6 (60%) patients respectively. Six (60%) patients had positive culture of blood or respiratory secretions with *Pseudomonas aeruginosa* identified as the causative agent. All patients (100%) had more than one phenotypic feature.

**Table 1:** Phenotypic features, values of the sweat tests and the genotypes of the cases examined in this study

Case number	Age at diagnosis	Consan-guinity	Phenotype	Sweat test (mmol/L)	Genetic studies	*CFTR* Genotype
Case 1	3.5 months	no	Recurrent LRTI (left lobe collapse), FTT, Pseudo-Bartter syndrome	NaCl = 116 Cl^−^ = not done	ARMS-PCR and RFLP, Gene sequencing	ƍf508/[C.1282C > G; C.2738A > G]
Case 2	6.5 months	no	Recurrent LRTI, right upper lobe collapse with basal emphysema. nasopharyngeal aspirate yielded *Pseudomonas*, steatorrhoea*,* FTT	sweat volume was inadequate	ARMS-PCR and RFLP	ƍF508/unknown
Case 3	6 months	no	Recurrent LRTI, *Pseudomonas* septicaemia, FTT, hypokalaemia	NaCl = 98 Cl^−^ = not done	ARMS-PCR and RFLP	Negative for the common 8 mutations in India
Case 4	12 months	no	Recurrent LRTI, Pseudo-Bartter syndrome, nasopharyngeal aspirate yielded mixed growth of *Pseudomonas* and coliform, FTT	NaCl = 72 Cl^−^ = not done	ARMS-PCR and RFLP	ƍf508/unknown
Case 5	3 years	no	Pseudo-Bartter syndrome, recurrent LRTI, FTT	NaCl = 126 Cl^−^ = 90	ARMS-PCR and RFLP	Negative for the common 8 mutations in India
Case 6	3.5 months	yes	Chronic LRTI, needed mechanical ventilation, FTT, respiratory secretions yielded *Pseudomonas*	NaCl = 130 Cl^−^ = 103	Gene sequencing	1161delC/1161delC
Case 7	2.5 months	no	Recurrent LRTI, cyanosis and oxygen dependancy, hyponatraemia, FTT	NaCl = 105 Cl^−^ = 83	ARMS-PCR and RFLP	Negative for the common 8 mutations in India
Case 8	10 years	no	Pseudo-Bartter syndrome, recurrent LRTI, gall stones, sputum culture is positive for *Pseudomonas*	NaCl = 107 Cl^−^ = 96	ARMS-PCR and RFLP, Gene sequencing	185 + 1G- > T/[C.1282C > G; C.2738A > G]
Case 9	22 years	no	Bronchiectasis, Pseudo-Bartter syndrome, BMI = 16.6 Kg/m^−2^ (underweight = < 18.5 Kg/m^− 2^)	NaCl = 82 Cl^−^ = 58	Gene sequencing	V456A/ V456A
Case 10	4 months	no	FTT, steatorrhoea, chronic LRTI, sputum positive for *Pseudomonas*, Pseudo-Bartter syndrome	NaCl = 63 Cl^−^ = 37	Gene sequencing	2184insA/CFTRdele4–11

Among 10 cases studied 7 (70%) were girls. Only one patient (10%) had consanguineous parents. The age at diagnosis ranges from 2.5 months to 22 years and the mean age at diagnosis is 46 months (~ 4 years).

### Identified mutations

Altogether 7 CF-causing mutations were identified in 10 patients. Out of the common 8 *CFTR* mutations in India, only delta F508 and 1161delC mutations were identified on 3 (15%) and 2 (10%) chromosomes respectively. The other six common mutations in India (1525-1G-A, 1792insA, R117H, S549 N, R553X and G551D) were not identified in our clinical samples. Gene sequencing identified all the mutations in tested samples; [C.1282C > G; C.2738A > G], V456A, C.53 + 1G > C, 2184insA and a deletion encompassing exons 4 to 11.

## Discussion and conclusions

To date, over 2000 variants in *CFTR* gene have been identified but not all of them cause the disease. Some are involved in milder *CFTR* related disease and there are a number of polymorphisms which are not associated with any clinical disease. Over 370 *CFTR* variants are annotated on the most recent list updated in Clinical and Functional Translation of CFTR (CFTR2) project. This information is derived from over 89,000 patients with specific *CFTR* variants from United States, Canada and Europe. Over 300 CF-causing variants are identified [17].

The most common phenotypic features in this sample of symptomatic patients were recurrent or persistent LRTIs, FTT and Pseudo-Bartter syndrome. Pseudo-Bartter syndrome was evident by hypokalaemic hypochloraemic metabolic alkalosis associated with hyponatraemia. Associated non-pulmonary complications like unexplained FTT and Pseudo-Bartter syndrome in a child or a young patient with recurrent or chronic pulmonary infections should trigger screening for CF [18, 19]. Majority presented early in life and had more severe CF phenotype except case 9 with less severe phenotype which is consistent with the hypothesis that the *CFTR* genotype affects the age at diagnosis [20].

All genetic variants identified in our patients except [C.1282C > G; C.2738A > G] are already annotated as CF-causing in CFTR2. It is a limitation of the study that mutations in some chromosomes remain uncharacterized due to inefficiency of the used mutation panel in detecting regional mutations in Sri Lanka that would have been detected by performing gene sequencing. Allele frequency of each variant in CFTR2 database is shown in Table 2. The frequency of the commonest mutation, delta F508 in CFTR2 is nearly 70% [17]. The study indicates that ΔF508 with allele frequency of 15% in this clinical sample is likely to be the commonest variant in Sri Lankan patients as well. Allele frequency of ΔF508 in Indian subcontinent is significantly lower than that observed in Caucasian population [21]. All the other mutations detected in Sri Lankan patients are rare mutations.

**Table 2:** Characteristics of CF-causing genetic variants detected in Sri Lankan patients, annotated by CFTR2

Variant cDNA name (ordered 5′ to 3′)	Legacy name	Allele frequency in CFTR2	Variant final determination	Case number
c.1521_1523delCTT	F508del	0.69744	CF-causing	Case 1 Case 2 Case 4
c.1029delC	1161delC	0.00015	CF-causing	Case 6
c.53 + 1G > T	185 + 1G- > T	0.00006	CF-causing	Case 8
c.1367 T > C	V456A	0.00019	CF-causing	Case 9
c.2052_2053insA	2184insA	0.00232	CF-causing	Case 10
c.(273 + 1_274–1)_(1679 + 1_1680–1)del	CFTRdele4–11	0.00002	CF-causing	Case 10
c.1393-1G > A	1525-1G- > A	0.00051	CF-causing	Reference [40]

The Department of Chemical Pathology has previously reported case 1 and case 8 with [C.1282C > G; C.2738A > G]. Both cases were born to non-consanguineous parents. In both cases the C.1282C > G and C.2738A > G variants are “in cis” encoding [(Leu428val;Tyr913Cys)] [22, 23]. The C.2738A > G variant has also been reported from France, Italy, Spain and Argentina. In silico prediction indicates that this single nucleotide variant is likely to be pathogenic. Replacement of tyrosine by cysteine at codon 913 of CFTR protein may result in abnormal disulfide bridge formation rendering this missense mutation deleterious. Case 1 (Table 1) supports the previously described fact that this change occurs on the chromosome that carries the non-delta F508 allele [24–26]. Even though this variant has not been evaluated by CFTR2 to date, these two cases in the present study along with previously reported cases with severe phenotype and high sweat sodium chloride and chloride concentrations provide clinical evidence that this novel mutation is CF-causing. 2184insA observed in case 10 is the second most common mutation in Western Ukraine. Insertion of adenine in position results in frameshift and production of an abnormal truncated CFTR protein. This deleterious mutation results in a severe phenotype with high sweat chloride concentrations, severe chronic lung disease, severe FTT associated with pancreatic insufficiency evident by low fecal elastase levels [27]. 2184insA mutation is reported from European countries like Czech Hungary, Poland, Germany, Slovakia, Bulgaria and also from Iran [28–34]. The deletion encompassing exon 4 to 11 of the *CFTR* gene has been previously reported in a child with European ancestry who showed a severe disease phenotype [35]. 185 + 1G > T mutation observed in case 8 has also been reported from Czech and Hungary [28, 29]. Presence of these European mutations in our population could be a result of historical Portuguese and Dutch conquests in Sri Lanka. The descendants of the inter-marriages between the Portuguese, Dutch and the Sinhalese and Tamils may inherit some genetic variances originated in Europe.

V456A is a missense mutation in exon 9 of *CFTR* which results in change of valine to an alanine in CRTR protein. Case 9 of present study and two other previously reported female cases from India and Pakistan who were homozygous for this mutation had intermediate sweat chloride concentrations with mild to moderate phenotype leading to delayed diagnosis of CF [36]. 1161delC mutation has been previously reported from Indian, Pakistani and Bahraini patients. This single base deletion of a cytosine at position 1161 of the *CFTR* gene results in a chain termination in codon 343 [37, 38]. This deleterious mutation in homozygous form resulted in severe pulmonary manifestations in case 6 of our study. The homozygous form was also observed in a Pakistani girl who died at age of 14 months [39]. A *CFTR* mutation in a child born to unrelated Sri Lankan parents who have immigrated to Canada was reported in 2003. This child presented at age of 3 months with clinical features of a full-blown kwashiorkor. Routine genotype analysis failed to detect *CFTR* gene mutations on either allele. Extensive genetic analysis revealed a splice acceptor variant (1525–1 G- > A). The mutation on the second allele was not identified [40]. The Canadian study which detected this case suggests that the prevalence and natural history of CF in South Asians is similar to that among individuals of European origin [41]. This mutation has been reported in Pakistani and Afghan patients who showed severe phenotypic features [38, 42, 43]. Nevertheless a Serbian patient homozygous for the same mutation showed a mild form of CF [44].

The geographic distribution of CF-causing mutations observed and reviewed in this study reflects the historical population migration from Europe and Indian subcontinent. A number of previous studies also suggest a significant genetic flow from rest of the Indian subcontinent to Sri Lanka [11–14]. The panel of common Indian mutations was ineffective to screen mutations in Sri Lankans due to heterogeneity of mutations in Sri Lankan population with significant proportion of mutations of European origin.

The World Health Organization has annotated Sri Lanka as “CF not recorded”. But these cases witness the fact that CF has been largely under-diagnosed in Sri Lanka. This may be partly due to lack of familiarity with CF among health care professionals. Moreover, the developing countries like Sri Lanka share common problems like, lack of neonatal screening programs for CF, reduced life expectancy in diagnosed patients relative to developed countries, poor availability of necessary drugs and a lack of CF services [45, 46]. All these socio-economic factors along with rare genotypes may contribute to worse disease outcome in Asian children compared to Caucasian counterparts [47]. A better awareness of CF and the increasing availability of diagnostic tests such as sweat chloride test and/or DNA tests will lead to the early identification of CF, higher case detection and better outcome [48].

Detection rate for a mutation panel depends on the carrier rate and mutation frequencies of a particular ethnic group. Considering the heterogeneous nature of mutations detected in this study, we can speculate that the remaining uncharacterized mutations might also be rare but can probably be detected by performing gene sequencing. The common mutations should be identified by sequencing the entire *CFTR* gene in adequate number of CF patients in order to design a mutation panel for common regional mutations. However gene sequencing is expensive and not readily available in Sri Lanka. But the sweat test can accurately diagnose more than 98% of cases of CF. Therefore, the reliable sweat test which is available in the country remains the most suitable and reliable approach in diagnosing CF in Sri Lanka. The study also highlights the importance of considering the diagnosis of CF in children and young adults presenting with persistent respiratory tract infections associated with severe malnutrition and Pseudo-Bartter syndrome.

## Abbreviations

ARMS: Amplification refractory mutation system
CF: Cystic fibrosis
CFF: Cystic fibrosis foundation
CFTR: Cystic fibrosis transmembrane conductance regulator
CFTR2: Clinical and Functional Translation of CFTR
CLSI: Clinical Laboratory Standards Institute
FTT: Failure to thrive
LRTI: Lower respiratory tract infection
MLPA: Multiplex ligation-dependent probe amplification
NBS: Newborn screening
PCR: Polymerase chain reaction
RFLP: Restriction fragment length polymorphism
RIQAS: Randox International Quality Assessment Scheme
